# The effect of incentives on residents’ board certification passage rate

**DOI:** 10.5116/ijme.5b6b.2192

**Published:** 2018-08-17

**Authors:** Peter Nguyen, Rotendo Nkomo, Rosibel Arcia, Kenneth Soyemi

**Affiliations:** 1 Departments of Pediatrics, Cook County Health, and Hospitals System, John H. Stroger Jr Hospital, USA; 2 Department of Emergency Medicine, Cook County Health, and Hospitals System, John H. Stroger Jr Hospital, USA

**Keywords:** Effect of incentives, residents’ board certification, passage rate, USA

## Introduction

Board certification after completing a pediatric residency is a significant milestone for all pediatric residents (individuals enrolled in an Accreditation Council for Graduate Medical Education (ACGME) accredited residency program).^1^ The process of board certification is based on the six competencies laid out by the ACGME culminating in the American Board of Pediatrics (ABP) certifying examination (CE) which was designed to evaluate an examinees ability for critical thinking and medical knowledge. Per the ABP, to complete board certification, a minimum scaled score of 180 out of 300 is required. Previous but limited studies have shown three significant predictors of successful board certification at the individual and training program-levels. The United States Medical Licensing Exam (USMLE) is a three-step examination for medical licensure in the US. The USMLE assesses a physician's ability to apply knowledge, concepts, and principles, and to demonstrate fundamental patient-centered skills, that are important in health and disease and that constitute the basis of safe and effective patient care.^2^ Welch and colleagues showed that both USMLE step exams predicted first-time pediatric board pass. Furthermore, they also concluded that results from step 2 were a stronger predictor of first-time pediatric board pass compared with that of step 1.^3^ At the start of every academic year in July, the American Board of Pediatrics holds their in-training exam (ITE) to assess the resident competency to achieve board certification. Although ITE scores have been used as validated predictors of BC in other medical specialties, that has not been the case in pediatrics until 2008 when Althouse and McGuiness showed that ITE scores of 3rd-year residents (PGY-3) have the most reliable predictive power of achieving board certification.^4^ Limited data exist on the effect of class size on the effect class size on board certification, however, two studies were done by Falcone and colleagues in both pediatric and internal medicine residencies respectively demonstrated larger class size as an independent predictor of board certification. They determined that the critical size cut-off for both pediatric and internal medicine residencies as 12 residents per year in order to achieve board certification.^5^^,^^6^ They further hypothesized that larger residency programs tend to have more resources to invest into the curriculum, hire highly trained faculty, and provide performance incentives. At approximately $2300 (circa 2018), the ABP CE is the most expensive of all the primary care specialties. It will not be surprising that without some form of assistance this fee can impact a resident’s financial status and consequently a resident’s ability to focus on examination preparation. We looked to examine the relationship between board certification and financial incentivization. After our program’s implementation of a tier-reward system in 2015, we sought to determine whether financial incentive provision had an impact on successful board certification completion on the first attempt.

### Overview of Tier-Reward System

We analyzed the five-year first-time attempt at board certification and observed that our residents average passing percent (69%) was lower when compared with the national average (85%) from 2011 through 2015. The percent successful first-time attempt board certification passage is a tool most residency programs use to recruit quality residents to residency training programs; our core faculty recognized that improving our percent of successful first-time board certification to meet national average was an important goal. An attending physician in our program served as a benefactor of the board examination financing funds; however, the core faculty decided to apply a tier-reward system in which reimbursement was based on the first-time attempt board certification to promote accountability amongst the residents instead of just merely providing the necessary funds. Once a resident was board eligible (BE), registered for the board exam, half of the exam fee was automatically reimbursed. The remaining balance would be refunded according to the percentage of the class pass rate (i.e., 70% board pass rate=70% of remaining balance reimbursed). To the best of our knowledge, we are not aware of any program that incentives residents to register and pass board certification  examinations; similar modified token economies have been used extensively in psychiatric patients to alter positive behaviors however we failed to find any evidence of this being as part of graduate medical education. Prior to implementation, we completed an informal survey of the PGY-3 class on their motivations for board certification to which the top 2 responses were; financial gain and requirement for fellowship and practice. Since the implementation of the tier-reward system, we have noted significant improvement in our board pass rate albeit a small sample size. From our previous 5-year running average of 69%, average first-time board pass rate improved to 95% with other factors including class size and ITE scores remaining constant.

## Conclusions

Although the exact mechanism of why financially incentivizing our resident's improved first-time attempt board certification pass-rates remains unknown, we postulate that the tier-reward system creates an increased sense of accountability and reduces financial induced stress amongst the residents which allow residents to focus and concentrate. A post certification informal survey among successful test-takers, it was interesting to note that the motivation for passing shifted from an individual drive, i.e., personal financial gain, to a shared group drive. The underlying motivation for the increased accountability we believe stems from the fear of being “the one who cost the class 10%”.  This pilot project generates an interesting point because it demonstrated that resident motivation was driven less by financial compensation as it was by resident camaraderie.  It is understandable that not all pediatric programs, especially smaller ones, will have these financial resources in order to generate a similar tier-reward system however it has yielded promising results thus far and should continue to be investigated.

### Conflict of Interest

The authors declare that they have no conflict of interest.
